# Global Meningococcal Initiative: guidelines for diagnosis and confirmation of invasive meningococcal disease

**DOI:** 10.1017/S0950268816001308

**Published:** 2016-06-30

**Authors:** J. A. VÁZQUEZ, M. K. TAHA, J. FINDLOW, S. GUPTA, R. BORROW

**Affiliations:** 1 National Reference Laboratory for Meningococci, Institute of Health Carlos III, Majadahonda Spain; 2 Institut Pasteur, Invasive Bacterial Infections Unit and National Reference Centre for Meningococci, Paris, France; 3 Vaccine Evaluation Unit, Public Health England, Manchester Royal Infirmary, Manchester, UK; 4 Department of Microbiology, National Centre for Disease Control, New Delhi, India

**Keywords:** Diagnosis, GMI, meningitis, meningococcal disease

## Abstract

The Global Meningococcal Initiative (GMI) is an international group of scientists and clinicians with recognized expertise in meningococcal disease including microbiology, immunology, epidemiology, public health and vaccinology. The GMI was established to promote the global prevention of meningococcal disease through education, research and international cooperation. The GMI held its second summit meeting in 2013 to discuss the different aspects of existing meningococcal immunization programmes and surveillance systems. Laboratory confirmation and characterization were identified as essential for informing evidence-based vaccine implementation decisions. The relative merits of different confirmatory methodologies and their applications in different resource settings were a key component of the discussions. This paper summarizes the salient issues discussed, with special emphasis on the recommendations made and any deficiencies that were identified.

## INTRODUCTION

The Global Meningococcal Initiative (GMI) is an independent, expert forum of scientists, clinicians and public health officials from across the world. GMI members have recognized expertise in meningococcal disease (MD), including aspects related to microbiology, immunology, epidemiology, public health and vaccinology. The Initiative aims to promote the global prevention of MD through education, research and international cooperation [1].

From data presented at a GMI summit meeting in 2010, it was apparent that meningococcal epidemiology is continuously changing and that there are significant regional and temporal differences. It was therefore recommended that country-/region-specific vaccination recommendations – as opposed to global guidelines – should be developed [1]. Consequently, subsequent GMI meetings have been regionally focused [2, 3], but the GMI has maintained its general principles regarding the promotion of laboratory-based surveillance as the main tool for understanding the true burden of the disease, as well as evaluating meningococcal immunization programmes, particularly for resource-poor countries [1].

The GMI held its second summit meeting in 2013 to discuss various aspects of existing meningococcal immunization surveillance programmes, with a focus on the areas where knowledge and experience are lacking. The relative merits of different confirmatory and characterization methodologies, and their application into different resource settings, were a key component of the discussions. This paper summarizes the salient issues discussed at this meeting and the subsequent discussions.

## SAFETY CONSIDERATIONS WHEN HANDLING MENINGOCOCCI IN THE LABORATORY

*Neisseria meningitidis* is classified as a hazard group 2 organism and should to be handled under containment level 2 or higher. Laboratory staff will be at risk when handling live meningococcal cultures and clinical samples containing viable meningococci if the appropriate safety controls are not maintained [4].

### Laboratory staff

Staff must be informed and trained on the biological risks of working with meningococci and informed on the signs and symptoms of MD. It is of paramount importance that all standard operating procedures and risk assessments are implemented and followed. Vaccination of staff with available meningococcal vaccines is strongly recommended and vaccinations against other agents (including hepatitis B) should be kept up to date.

### Laboratory

The main safety risk is exposure to aerosols containing viable meningococci. It is therefore of paramount importance that any procedure that could result in the production of aerosols (e.g. handling liquid suspensions) is undertaken with appropriate containment and, where possible, the risk of aerosol production should be engineered out of the processes (by replacing liquid culture with solid culture, for example).

## CURRENT DIAGNOSIS, CONFIRMATION AND CHARACTERIZATION OF INVASIVE MENINGOCOCCAL DISEASE (IMD)

Although immediate antibiotic treatment should not be delayed upon suspicion of IMD, diagnosis requires rapid confirmation to enable reliable antibiotic treatment of patient and chemoprophylaxis among contacts. This should be followed by characterization to further inform contact management; for example, through immunization if the pathogen is found to be vaccine preventable.

### Diagnosis: clinical criteria

Early signs and symptoms are usually unspecific. Patients may present with headache, neck stiffness, fever, projectile vomiting and photophobia, also with lethargy or altered consciousness in more advanced disease [5]. Presence of a non-blanching haemorrhagic rash (purpura fulminans) is distinctive for IMD and usually prompts immediate antibiotic treatment. Although meningitis and sepsis are the two most frequent symptoms, other presentations may also be observed (e.g. arthritis and pericarditis). For example, the US Centers for Disease Control and Prevention (CDC) lists clinical purpura fulminans as the clinical criterion for suspected IMD [6], while the European Centre for Disease Prevention and Control (ECDC) clinical criteria include any person with at least one of the following: meningeal signs, haemorrhagic rash, septic shock or septic arthritis [7]. Australian guidance similarly gives the absence of evidence for other causes of clinical symptoms, combined with either clinically compatible disease including haemorrhagic rash or compatible disease, plus close contact with a confirmed case within the previous 60 days [8].

### Case confirmation

Meningococci are Gram-negative diplococci and are found extracellularly or intracellularly in peripheral mononuclear leukocytes. The initial laboratory screening of a potential IMD case can incorporate a number of widely available and well-established laboratory evaluations; samples may be taken from cerebrospinal fluid (CSF), peripheral blood or skin lesions or, when symptoms indicate, from synovial, pleural or pericardial fluid [6]. For example, cytology using CSF or peripheral blood smears, detection of bacterial polysaccharides, as well as detection of blood markers such as C-reactive protein, are methods available in most hospital laboratories and often enable differentiation between viral or bacterial meningitis.

Recommendations for the use of specific methods for diagnosis and confirmation will depend largely on the case definition employed. For example, in some countries, the presence of Gram-negative diplococci in a CSF or blood smear – or from another normally sterile site – is considered confirmatory, while in others, blood smears from skin lesions are also noted as confirmatory [6–8]. Antigen detection by immunohistochemistry is also considered confirmatory in some countries [6, 7].

#### Microbiological methods

The timing of specimen collection is crucial for successful processing of *N. meningitidis* culture [9]. False-negative results may result from early antibiotic treatment and incorrect handling or culturing of samples [9]. Growth is optimal at 35–37 °C in ~5% CO_2_, on blood agar or chocolate agar plates [9, 10].

Detection of soluble bacterial polysaccharides in CSF by latex agglutination is widely used to confirm *N. meningitidis* infection and can provide some typing information. It requires no specialized facilities or training (apart of centrifugation and reagents refrigeration), although timing of specimen collection is an issue and has been associated with false negatives; moreover, results are variable when used for direct detection in clinical samples such as CSF, with sensitivities ranging between 32% and 96% [11], particularly in capsular group B cases. Latex agglutination is also very effective in identifying capsular groups A, B, C, W and Y from culture.

*N. meningitidis* meningococci show a positive result in Kovac's oxidase test. This test determines the presence of cytochrome oxidase, i.e. presence of an organism containing cytochrome *c* as part of its respiratory chain – this could be *N. meningitidis* but also other bacterial organisms. As such, carbohydrate utilization tests are then used to validate the presence of *N. meningitidis* further [10]. Gram staining costs relatively little and interpretation of the test results requires only minimal resources and staff training. It is usually considered to be highly specific, but is of low sensitivity, particularly in cases with low bacterial load or where early antibiotic treatment has been instigated; it must also be performed in a relatively short period after collection, which is not always feasible where hospitals lack 24-h laboratory facilities. Matrix-assisted laser desorption ionization–time-of-flight mass spectrometry has also been described for the routine identification of microorganisms; however, improvement of meningococcal identification is required [12].

In cases with clinically suspicious and negative culture (including in close contacts), nasopharyngeal swabs can be utilized as they can yield meningococci in up to 50% of instances [13] and can be used to identify and characterize the organism [14]. Although the isolation of meningococci from the nasopharynx in a patient is not considered confirmatory, concordance between nasopharyngeal and invasive isolates has been found in 97% of cases [13].

#### Rapid diagnostic tests

Rapid testing using dipstick diagnostic tests is now becoming available and preliminary in-field data appear to be promising [15]. Test kits are designed to be used by non-specialized staff with minimal training in basic facilities. Thus far, tests have been developed for capsular groups A, C, W, Y and X [16, 17], and the GMI recommends that tests for additional capsular groups are developed.

#### Polymerase chain reaction (PCR) methods

Compared to conventional culture-based methods, PCR-based methods are not at risk from loss of viable organisms through early antibiotic treatment and sample processing [18, 19]. The production of false positives due to contamination, however, should be avoided by strict adherence to procedural guidelines. The general trend has been to include PCR as confirmatory in case definitions globally, although there is still some level of divergence on this point. For example, in the European Union, 25% of cases are confirmed by PCR alone, and in the UK and Ireland, this rises to ~50% [20]; while in the United States, the CDC only recently included PCR as confirmatory in its case definition [6]. There is as yet no international consensus on their use, although PCR methods incorporating a number of targets have been developed (such as *ctrA, porA, crgA*, 16S rDNA, *sodC* and *cnl*) [21–25].

The GMI noted that either regional/provincial or centralized reference facilities could be suitable for implementing PCR, providing that results are rapidly available (i.e. within hours). However, although the cost of PCR has been decreasing, its introduction in many countries is still hindered by resource constraints and lack of trained staff; to date, many countries still have not implemented PCR. Finally, despite adoption of PCR, culture is still required to recover viable strains for further characterization and to aid vaccine formulation. Hence, the GMI recommends that confirmatory PCR be instituted wherever possible, but without replacing culture or other methods. This is likely to result in a more detailed understanding of the real burden of disease in many countries.

### Characterization and surveillance

#### Serogrouping

Cultured isolates should be systematically serogrouped. The ability of reference laboratories to serogroup *N. meningitidis* is important as it enables public health authorities to identify outbreaks that are controllable by vaccination campaigns, recognize which serogroups are the cause of sporadic disease and detect the emergence of new serogroups. However, detecting the emergence of new outbreak strains does require additional characterization [10].

#### PCR and molecular genetic methods

Although PCR may be unavailable in remote areas or resource-poor settings, shipping samples to a central laboratory with PCR facilities could enable enhanced surveillance compared to microbiological and serogrouping techniques alone.

In recent years, new PCR and DNA sequencing methodologies have enhanced the characterization of *N. meningitidis* strains [26]. Multilocus sequence typing (MLST), involving characterization of short fragments of several microbial genes, has been utilized in numerous laboratories for successful characterization of isolates and particularly in global surveillance [27]. DNA sequencing methodologies have been used for molecular determiniation of sensitivity to antimicrobials through identification of allelic variants of specific genes and sensitivity/resistance to the corresponding antibiotics [28]. Specific antigen-sequence typing (AST) to characterize the variable regions of *N. meningitidis*, such as the antigen-encoding genes *porA* and *fetA*, can enable rapid investigation and characterization of antigenic variants as an aid for vaccine development or selection [29]. As whole-genome sequencing (WGS) technology becomes more widely available it is likely to supersede MLST and AST, enabling even more detailed strain characterization [26].

#### Levels of characterization

There was a consensus that characterization of meningococcal isolates should begin as soon as a case has been confirmed. Basic and advanced levels of characterization can be defined as follows:

1. Basic characterization information:
2. Capsular group identification (e.g. by dipstick testing, latex agglutination or PCR).
3. *PorA* and *fetA* typing data (e.g. by PCR, AST or WGS) [29].
4. Antibiotic susceptibility testing, including a minimum of antimicrobial agents to be tested: two of those used in chemoprophylaxis (rifampicin and quinolones) and two for clinical treatment (penicillin and ceftriaxone), with chloramphenicol included where other agents are not readily available.
5. Advanced characterization information:
6. Vaccine antigen variant definition (*fHbp, NHBA* and *NadA*) (e.g. by AST or WGS) [30].
7. Molecular population biology – MLST is currently utilized for this, although WGS will most probably become the established method [31].
8. Vaccine antigen expression evaluation using Meningococcal Antigen Typing System [32, 33] or flow cytometry [34] or other similar methodology developed in house.
9. Antibiotic susceptibility analysis (e.g. by PCR or WGS) for *penA* (penicillin G), *rpoB* (rifampicin) and *gyrA* (ciprofloxacin) [27, 35–37].

Most of the methodologies listed were of significant value when assessing the impact of interventions of conjugate vaccine against capsular group C and subsequently with quadrivalent conjugate vaccines or conjugate vaccine against capsular group A in the African meningitis belt [38]. The GMI therefore considered that it would be of great interest to use such tools to evaluate the impact of new sub-capsular vaccines for the prevention of capsular group B and recommends that a laboratory or regional laboratories with facilities able to undertake these methods be identified.

## SUMMARY AND CONCLUSIONS

Globally, infrastructure and facilities dictate how MD is confirmed and the level of surveillance undertaken. Laboratory confirmation is essential for informing evidence-based vaccine implementation decisions and many techniques are available, including microscopy, latex agglutination and PCR. The sensitivity and specificity of confirmation techniques can be affected by sample type, sampling procedures and antibiotic use. Most of the confirmation techniques provide results that form the basis of surveillance and are critical in aiding decisions about vaccine interventions. There was a general agreement, therefore, that, regardless of the approach employed for confirmation and further characterization, regular participation in internal–external quality-assurance schemes was essential to ensure optimal performance of these methods. Dipstick tests specific for A, C, W, Y and X capsular groups show promise for rapid diagnosis and DNA-based technologies, such as WGS, are likely to gain in importance for surveillance. Culture, however, remains the gold standard in confirmation and characterization, as it maintains the isolate for further characterization.
